# Effectiveness of Dietary Interventions in the Treatment of Endometriosis: a Systematic Review

**DOI:** 10.1007/s43032-020-00418-w

**Published:** 2021-03-24

**Authors:** Konstantinos Nirgianakis, Katharina Egger, Dimitrios R. Kalaitzopoulos, Susanne Lanz, Lia Bally, Michael D. Mueller

**Affiliations:** 1 Department of Obstetrics and Gynecology, University Hospital and University of Bern, Friedbühlstrasse 19, 3010 Bern, Switzerland; 2 Medical University of Vienna, Vienna, Austria; 3 Department of Obstetrics and Gynecology, Cantonal Hospital Schaffhausen, Geissbergstrasse 81, 8208 Schaffhausen, Switzerland; 4 Department of Diabetes, Endocrinology, Clinical Nutrition & Metabolism, University Hospital and University of Bern, Freiburgstrasse 15, 3010 Bern, Switzerland

**Keywords:** Dietary intervention, Alternative treatment, Complementary treatment, Nutrition, Diet

## Abstract

A patients’ increasing interest in dietary modifications as a possible complementary or alternative treatment of endometriosis is observed. Unfortunately, the therapeutic potential of dietary interventions is unclear and to date no guidelines to assist physicians on this topic exist. The aim of this study, therefore, was to systematically review the existing studies on the effect of dietary interventions on endometriosis. An electronic-based search was performed in MEDLINE and COCHRANE. We included human and animal studies that evaluated a dietary intervention on endometriosis-associated symptoms or other health outcomes. Studies were identified and coded using standard criteria, and the risk of bias was assessed with established tools relevant to the study design. We identified nine human and 12 animal studies. Out of the nine human studies, two were randomized controlled trials, two controlled studies, four uncontrolled before-after studies, and one qualitative study. All of them assessed a different dietary intervention, which could be classified in one of the following principle models: supplementation with selected dietary components, exclusion of selected dietary components, and complete diet modification. Most of the studies reported a positive effect on endometriosis; they were however characterized by moderate or high-risk bias possibly due to the challenges of conducting dietary intervention trials. According to the available level of evidence, we suggest an evidence-based clinical approach for physicians to use during consultations with their patients. Further well-designed randomized controlled trials are needed to accurately determine the short-term and long-term effectiveness and safety of different dietary interventions.

## Introduction

### Endometriosis and Current Therapeutic Limitations

Endometriosis, characterized by the growth of endometrial-like tissue outside the uterine cavity, is an estrogen-dependent chronic inflammatory highly prevalent gynecological disorder of reproductive-aged women worldwide [1–3]. It is a significantly heterogeneous disease, both in phenotype and clinical outcomes that vary from no symptoms to severe pain and/or subfertility leading to a significant reduction in quality of life [4, 5]. Moreover, the economic impact is substantial, as chronic and debilitating pain from endometriosis may hinder work productivity, while infertility can cause major psychosocial and financial strain to affected women and their partners [6].

Treatment options are either hormonal-based therapies or laparoscopic surgical excision of the endometriotic lesions. According to current guidelines, endometriosis-related pain should be empirically treated with adequate analgesia and combined oral contraceptives or progestins prior to a definitive diagnosis [7]. Unfortunately, adjuvant hormonal therapy is often accompanied by significant, unwanted side effects and in about 30% of the patients a non-adequate reduction in endometriosis-associated pain [8]. In another recent study, 45.4% of the patients have been reported to be unsatisfied with their medical treatment [9] while high treatment discontinuation rates have been observed [10]. As a result, many patients will undergo laparoscopic excision of endometriosis, which is associated with decreased overall pain, both at 6 and 12 months after surgery as well as fertility improvement in some cases [11, 12]. However, even after a complete removal of endometriotic lesions a high proportion of patients will require additional surgery due to endometriosis recurrence with total recurrence rates of 21.5% and 40–50% at 2 and 5 years, respectively [13–17]. To avoid disease, recurrence postoperative adjuvant hormonal therapy is advised until planning a pregnancy [18, 19].

Given the above and considering that these therapeutic options are non-curative and may not align with women’s reproductive goals, it becomes apparent that there is an unmet need for improved treatment of endometriosis and associated symptoms.

### Nutrition and Risk of Endometriosis: Dietary Intervention Possible?

Nutrition is widely recognized as a prognostic and modifiable factor related to morbidity and life expectancy [20–23]. Several observational studies have investigated certain nutrition habits as risk factors for endometriosis [24–38]. Women with endometriosis seem to consume fewer vegetables, omega-3 polyunsaturated fatty acids, and dairy products and more red meat, coffee, and trans fats; but these findings could not be consistently replicated [39]. A recent review summarizing the linkage between diet end endometriosis underlined the potential of anti-inflammatory components present in foods to mitigate endometriosis [40]. However, to date, no study has reviewed the therapeutic potential of dietary interventions in patients with already diagnosed endometriosis. As complementary therapies and self-management for women with endometriosis including dietary modifications become more widely popular, it is important to ensure their safety and effectiveness.

Our study, therefore, aimed to conduct a systematic review of the literature investigating the effect of dietary interventions on endometriosis. We intended to cover the literature on a number of sub-questions, including (a) if a certain subcategory of patients are more likely to benefit from a dietary intervention or (b) if specific dietary interventions ameliorate certain symptoms while others remain unchanged.

## Materials and Methods

This systematic review was prepared according to the Preferred Reporting Items for Systematic Reviews and Meta-Analyses (PRISMA-P Statement). The study protocol was registered in the Bern Open Repository and Information System (BORIS) of the University of Bern under the number 143339.

### Search Strategy

The electronic medical information database Medline was searched up to 30. April 2020 with the following search algorithm: (endometriosis) AND ((diet*) OR (nutrition) OR (fasting)). The same keywords were used to search the Cochrane library. No filter or limitation was applied. Reference lists from selected studies were manually scanned to identify any other relevant studies.

### Eligibility Criteria

Original studies (cohort, case-control, and randomized controlled studies) including women diagnosed with endometriosis or animals with induced endometriosis assessing a dietary intervention on endometriosis-associated symptoms or other health outcomes were included. Possible dietary interventions were either adherence to a specific diet or intake of dietary supplements. Studies that did not present the dietary patterns, case reports, and conference abstracts as well as studies including patients with dysmenorrhea without the diagnosis of endometriosis and in vitro studies were excluded.

### Outcomes

Changes in endometriosis-associated symptoms measured with pain scales or patient reported quality of life outcomes. Regression of endometriosis is either reported by imaging examinations or a decrease of endometriosis-associated biomarkers. Change of other relevant health outcomes.

### Data Extraction

Titles and abstracts were evaluated independently by two reviewers. Any discrepancy was resolved by consensus. Data extraction was performed by the same two reviewers. For each study, information on publication data, study design, population characteristics, type of dietary intervention, results, and limitations were extracted.

A narrative synthesis of the data was applied to compare the effects of different dietary interventions on the outcomes and a meta-analysis was initially planned.

### Risk of Bias Assessment

The different types of bias assessment were performed independently by more than one investigator (KN, KE, and DRK for the human studies and KE and DRK for the animal studies). Quality assessment for observational human studies was performed by the Quality in Prognostic Studies (QUIPS) tool and for animal studies with the SYRCLE’s risk of bias tool (doi.org/10.1186/1471-2288-14-43). Risk judgment was assessed using pre-specified study criteria. For randomized controlled trials, five criteria of the Cochrane risk-of-bias tool were used ((selection bias (random sequence generation and allocation concealment); performance bias (blinding of participants and personnel); detection bias (blinding of outcome assessors); attrition bias (incomplete outcome data); reporting bias (selective reporting)) [41, 42].

## Results

**Fig. 1:**
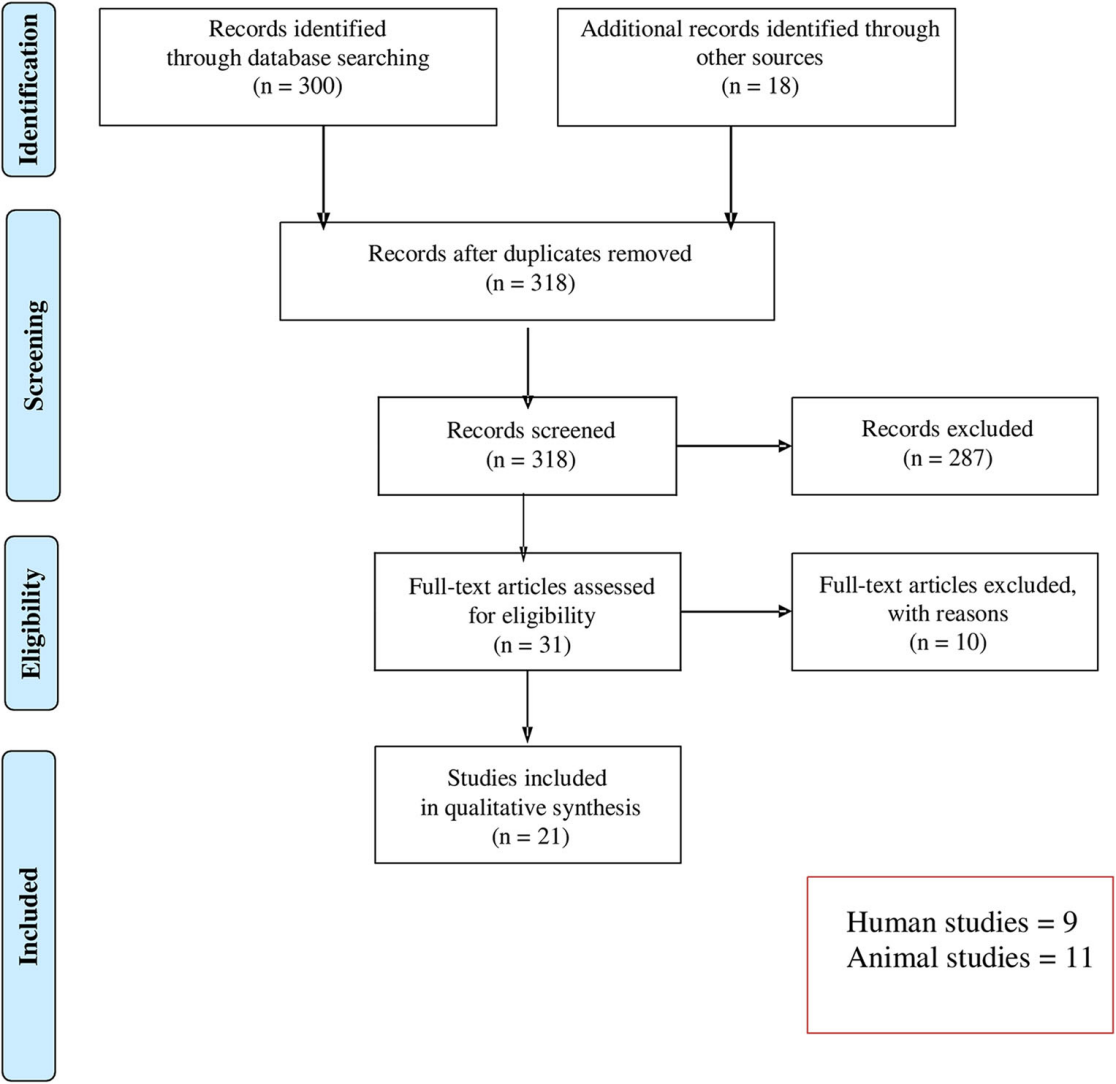
PRISMA flow diagram

A total of 318 studies were identified and screened for inclusion. After excluding 287 studies by reading the titles and abstracts, 31 studies were further assessed for eligibility of which 10 further articles were excluded due to study design and outcome measurements. At the end, nine human and 12 animal studies were included (Fig. 1).

### Human Studies

Out of the nine human studies, two were randomized controlled trials [43, 44], two prospective controlled studies [45, 46], two prospective [47, 48], and two retrospective [49, 50] uncontrolled before-after studies and one qualitative study [51]. The follow-up period of studies ranged from 4 weeks to 12 months. Studies were from Italy (*n* = 3), Austria (*n* = 1), Sweden (*n* = 1), Australia (*n* = 1) Mexico (*n* = 1), and Iran (*n* = 1).

Interventions comprised of supplementation of vitamin D [43]; supplementation of vitamins A, C, and E [46]; supplementation of omega-3/6; quercetin; vit B3; 5-methyltetrahydrofolate calcium salt, turmeric, and parthenium [45]; Mediterranean diet [48]; low-FODMAP diet [49]; low nickel diet [47]; gluten-free diet [50]; and individual diet changes [51]. The detailed characteristics of the human studies and the risk of bias assessment are presented in Tables 1, 2, and 3.

**Table 1:** Characteristics of the human studies

Author	Country	Study design	Intervention group	Control group	Dietary intervention	Outcomes	Results	Limitations
Almassinokiani et al., 2016 [43]	Iran	Double-blind randomized controlled trial on patients with persistent symptoms after laparoscopy	19	20	Supplementation of Vitamin D (50,000 iu/weekly) for 3 months and placebo diet	Severity of pelvic pain/ dysmenorrhea before laparoscopy, at 2. menses and after intervention measured by VAS (0–10)	No significant difference between effect of vitamin D and placebo	Small sample size
Borghini et al., 2020 [47]	Italy	Prospective uncontrolled before-after study on patients with endometriosis and gastrointestinal symptoms	47 included but 31 completed the study	-	Low-Ni diet for 3 months	- Dysmenorrhea, dyspareunia, and pelvic pain measured by VAS (1–10)—gastrointestinal symptoms measured by the Gastrointestinal Symptom Rating Scale (GSRS) questionnaire—nickel oral mucosa patch test	- 28/31 (90.3%) had a positive Nickel Oral Mucosa Patch Test—all gastrointestinal symptoms and dysmenorrhea, dyspareunia and pelvic pain showed a statistically significant decrease in intensity	- 14 out of 47 (29.8%) patients drop out- no intention to treat analysis
Marziali at al., 2012 [50]	Italy	Retrospective uncontrolled before-after study on patients with severe painful endometriosis-related symptoms	295	–	Gluten-free diet for 12 months	- Dysmenorrhea, non-menstrual pelvic pain, and deep dyspareunia measured by VAS (1–10)—quality of life	- 156/207 patients (75%) reported statistically significant change in painful symptoms (*P* < 0.005), 51/207 patients (25%) reported not improvement of symptoms- A considerable increase of scores for all domains of physical functioning, general health perception, vitality, social functioning and mental health was observed in all women (*P* < 0.005).	- At 2 weeks after starting the diet, 88 (30%) withdrew because of side effects as abdominal symptoms. No intention to treat analysis—VAS scores were not presented, the change in painful symptom not presented per type of pain (e.g., dysmenorrhea, non-menstrual pelvic pain, dyspareunia)
Mier-Cabrera J. et al., 2009 [46]	Mexico	Prospective case-control study on infertile patients with endometriosis rASRM I–II	35	37	Antioxidant diet by supplementation of Vitamin A,C and E or normal diet for 4 months	- Vitamin intake measured by Food Frequency Questionnaire (FFQ) for Mexican women—oxidative stress determination by measurement of total plasma lipid hydroperoxides (LHP) and malondialdehyde (MDA) –vitamins in serum, plasma, and leukocytes–superoxide dismutase (SOD) and glutathione peroxidase (GPx) activity in plasma	- Endometriosis patients had a lower intake of antioxidant vitamins compared to patients without endometriosis- Peripheral oxidative stress markers diminished, and antioxidant markers were enhanced after the dietary intervention	- The normal diet or the antioxidant diet was tailored according to each patient’s energy requirement - Lack of clinical outcomes
Moore et al., 2017 [49]	Australia	Retrospective uncontrolled before-after study on patients with irritable bowel syndrome with or without endometriosis	160 patients with irritable bowel syndrome; 59 of them of with endometriosis and 101 without	-	Low-FODMAP diet for 4 weeks	A greater than 50% reduction in abdominal symptoms. Symptoms were documented and the intensity measured by a 40-question tool and VAS.	A significantly higher proportion of patients with known endometriosis responded to the diet (*n* = 43, 72%) compared with those without (*n* = 49, 49%; *P* = 0.001) according to intention-to-treat (OR 3.11, 95% CI 1.5–6.2).	-Retrospective design-VAS scores not presented
Ott et al., 2012 [48]	Austria	Prospective uncontrolled before-after study on patients with persistent symptoms after laparoscopy	68	-	Mediterranean diet for 5 months	Changes in symptoms: general pain, condition, dysmenorrhea, dyspareunia, dyschezia measured by NAS (0–10)	Significant relief of general pain (NRS 4.2 ± 3.0 vs. 2.0 ± 2.3; *p* < 0.01), as well as an improvement in the general condition (NRS 6.7 ± 2.2 vs. 8.5 ± 1.7; *p* < 0.01)	- No control group—25 (36.8%) patients not adhered to the dietary regimen
Sesti et al. 2007 [44]	Italy	Randomized controlled trial on patients with endometriosis rASRM III-IV	37	1) 115 with placebo 2) 42 with GnRHa (tryptorelin or leuprolin) 3) 40 with continuous ethynilestradiol + gestoden	For 6 months, vitamins (B6, A, C, E), mineral salts (Ca, Mg, Se, Zn, Fe), lactic ferments, fish oil (Omega-3/6), which secured nutritional rate between 1600 and 2000 cal. A different dietary protocol was assigned according to the body mass index, the physical activity, and the job of each woman.	- Dysmenorrhea, non-menstrual pelvic pain, deep dyspareunia (VAS 0–10) at 12 months- Quality of life (SF-36 score) at 12 months	At 12 months’ follow-up, patients in the GnRHa or continuous COC groups reported less scores for dysmenorrhea than patients in the control group or dietary intervention. All treated groups reported less non-menstrual pain than the placebo group. Dyspareunia was more decreased in the placebo group though. Increase of scores for all domains of SF-36 was observed independently of the treatment allocation.	- 5 patients in the placebo group and 7 patients in the other groups who withdrew or lost to follow-up were excluded from the analysis (no intention to treat analysis)—outcomes were analyzed 12 months after randomization or 6 months after the end of treatments
Signorile et al., 2018 [45]	Italy	Prospective case-control study on rASRM stage IV patients	60 divided in two groups of 30	30	For 3 months, the first group treated with omega-3/6, quercetin, Vit B3, 5-methyltetrahydrofolate calcium salt, turmeric, parthenium. The second group treated with linseed oil and 5-methyltetrahydrofolate calcium salt. The third group with placebo.	- Intensity of headache, cystitis, muscular pain or fibromyalgia, irritable colon, dysmenorrhea, dyspareunia, chronic pelvic pain measured by VAS (0–100) before intervention and after 3 months—17b-estradiol—PGE2- CA-125	Significant reduction of the symptoms and the laboratory parameters in the first group	- Difference in VAS score and laboratory parameters not presented; just the number of patients with VAS > 5 before and after the dietary intervention. No clear description of the inclusion-exclusion criteria—no description of the period of recruitment
Vennberg Karlsson et al., 2020 [51]	Sweden	Qualitative interview study	12	-	Individual, voluntary dietary changes	Experiences of health after the dietary change reported during the interview	Increase in well-being and a decrease in symptoms	- Retrospective design—patient recruitment via endometriosis support forums—unclear dietary interventions—no objective measurement of pain symptoms

**Table 2:** Quality assessment of observational human studies

	Study participation	Study attrition	Prognostic factor measurement	Outcome measurement	Study confounding	Statistical analysis and reporting	Overall assessment
Borghini et al. [47]	Low bias	Moderate bias	Low bias	Low bias	Moderate bias	High bias	Moderate bias
Marziali et al. [50]	Low bias	Moderate bias	Low bias	Low bias	Moderate bias	High bias	High bias
Mier-Cabrera et al. [46]	Low bias	Low bias	Low bias	Low bias	High bias	Low bias	Moderate bias
Moore et al. [49]	Moderate bias	Low bias	Low bias	Moderate bias	High bias	High bias	Moderate bias
Ott et al. [48]	Low bias	Moderate bias	Moderate bias	Low bias	Moderate bias	Low bias	Moderate bias
Signorile et al. [45]	Moderate bias	High bias	Low bias	Low bias	Moderate bias	High bias	High bias
Vennberg et al. [51]	High bias	Moderate bias	High bias	High bias	High bias	High bias	High bias

**Table 3:** Quality assessment of RCT human studies

	Random sequence generation (selection bias)	Allocation concealment (selection bias)	Blinding of participants and personnel (performance bias)	Blinding of outcome assessment (detection bias)	Incomplete outcome data (attrition bias)	Selective reporting (reporting bias)
Almassinokiani et al. [43]	Low	Low	Low	Low	Low	Low
Sesti et al. [44]	Low	Low	Unclear	Unclear	High	Low

#### Uncontrolled Before-After Studies

One retrospective before-after study conducted in Italy and published in 2012 [50] investigated the effect of the gluten-free diet in 295 patients with moderate or severe endometriosis-associated symptoms. Two weeks after starting the diet, 88 (30%) patients withdrew due to side effects as abdominal symptoms. At 12 months of follow-up, 156 patients reported a statistically significant change from baseline in painful symptoms while 51 patients reported no improvement. Moreover, the scores in measurements of quality of life were significantly increased. The same group from Italy presented an oral communication in a congress in 2015 investigating retrospectively the gluten-free diet in a case-control study including 300 patients with endometriosis. Both groups were subjected to dienogest therapy (2 mg/day) while the case group received a gluten-free diet. The data showed a statistically significant improvement of pelvic pain in the group of patients with gluten-free diet. Since this study was never published in a peer-review journal, it was excluded from our further analysis [52].

In a retrospective analysis of a cohort study assessing the efficacy of a diet low in fermentable oligo-, di-, monosaccharides, and polyols (FODMAP) in 160 patients with irritable bowel syndrome, responses in patients with (*N* = 59) and without (*n* = 101) endometriosis were contrasted. FODMAPs are poorly absorbed, short-chain carbohydrates that are readily fermentable by bacteria. Their osmotic actions and gas production may cause intestinal luminal distension inducing pain and bloating in patients with visceral hypersensitivity with secondary effects on gut motility. Adherence to the diet was high in both groups with only four (7%) in the endometriosis group and ten (9%) in those without endometriosis showing insufficient compliance to assess efficacy. Forty-three (72%) of the patients with endometriosis experienced an improvement of symptoms over 50% after following this diet for 4 weeks. This was significantly higher than in the group of patients without endometriosis and represented a threefold increase in the likelihood of responding to the low-FODMAP diet compared to those without known endometriosis (OR 3.11, 95% CI 1.5–6.2) [49].

The prevalence of nickel allergic contact mucositis (Ni ACM) in patients with endometriosis and gastrointestinal disorders was evaluated in a recent Italian study. Forty-seven patients were included out of which 31 (70%) adhere to the suggested balanced low-Ni diet for 3 months. Twenty-eight out of the 31 patients studied (90.3%) showed oral mucosal patch test positive results. All gastrointestinal symptoms measured by the Gastrointestinal Symptom Rating Scale (GSRS) questionnaire (*p* < 0.05) as well as dysmenorrhea, dyspareunia, and pelvic pain measured by a numeric scale (0–10) (*p* < 0.005) showed a statistically significant decrease in intensity [47].

The influence of the Mediterranean diet on endometriosis-associated pain was examined in a single-arm study in Austria. Sixty-eight women with a previous laparoscopic diagnosis of endometriosis and postoperative endometriosis–associated pain were included. Patients had to adhere to a specific nutrition plan regimen including fresh vegetables, fruit, white meat, fish rich in fat, soy products, whole meal products, foods rich in magnesium, and cold-pressed oils. During the intervention, participants were asked to avoid sugary drinks, red meat, sweets, and animal fats. Pain degrees were specified on a Numeric Rating Scale (NRS). Twenty-five study participants (36.8%) did not adhere to the recommended dietary regimen due to pregnancy or change to classical treatment options. However, in an intention-to-treat analysis, all 68 patients were included. A significant relief of general pain, dysmenorrhea, dyspareunia, and dyschezia as well as an improvement in the general condition was found [48].

#### Controlled Studies

In a Mexican case-control study, 82 infertile patients with rASRM stages I–II endometriosis were randomly assigned either to a control group with normal diet (*n* = 37) or to a high antioxidant diet (HAD) group (*n* = 35) for 4 months according to each patient’s energy requirements. Adherence to the diet was high in both groups with 32 (91.4%) in the HAD and 34 (91.9%) in the normal diet group completing the study. An increase in the vitamin concentrations (serum retinol, alpha-tocopherol, leukocyte, and plasma ascorbate) and antioxidant enzyme activity (superoxide dismutase and glutathione peroxidase) as well as a decrease in oxidative stress markers (malondialdehyde and lipid hydroperoxides) were observed in the HAD group after 2 months of intervention. These phenomena were not observed in the control group. No clinical outcomes were examined in this study [46].

In an Italian placebo-controlled study, 90 laparoscopically diagnosed rASRM stage IV endometriosis patients were split into three groups. All of the patients had to increase the daily fiber intake by 20–30% and food containing omega 3. Moreover, the patients were asked to reduce the consumption of dairy products (by at least 30%), meat (by at least 50%), gluten-rich food, caffeine, alcohol, chocolate, saturated fat, butter, and margarine. Finally, the consumption of soy, aloe, and oats for all the observation was forbidden. Additionally to that, the first group was treated with a composition of dietary supplements (alpha linolenic acid (omega 3), linoleic acid (omega 6), quercetin, nicotinamide, 5-methyltetrahydrofolate calcium salt, titrated turmeric, and titrated parthenium)). The second group was treated with linseed oil and 5-methyltetrahydrofolate calcium salt and the third group with placebo. The study demonstrated a significant reduction of pain symptoms and serum levels of PGE2, 17b-estradiol, and CA-125 in the first group after 3 months. The authors concluded that the data indicated an anti-inflammatory action of the compounds that mimic the pharmacological action of the most common used drugs for the therapy of endometriosis [45].

In a randomized controlled trial conducted in Iran 40 patients with persistent pain at second menses after operative laparoscopy were double-blind randomized to receive either vitamin D (50,000 iu/weekly) or placebo for 12 weeks. The majority of the patients had rASRM stage III or IV endometriosis (87.5%). The severity of dysmenorrhea and pelvic pain was measured by the VAS test. No significant effect of vitamin D on the pain outcomes 24 weeks after laparoscopy was shown [43].

Another randomized controlled trial conducted in Italy [44] randomized patients with rASRM stages III–IV endometriosis at the time of postoperative control to placebo (*n* = 115), GnRHa (*n* = 40), continuous COC (*n* = 40), and nutritional supplements (vitamins (B6, A, C, E), mineral salts (Ca, Mg, Se, Zn, Fe), lactic ferments, fish oil (omega-3/6)) for 6 months. Dysmenorrhoea, non-menstrual pain, deep dyspareunia, and quality of life was measured preoperatively and at 12 months’ follow-up. Patients treated with hormonal suppression showed less visual analogue scale scores for dysmenorrhoea than patients of the other groups. Hormonal suppression therapy and dietary supplementation were equally effective in reducing non-menstrual pelvic pain. A significative decrease in dyspareunia scores was observed in the controls than in patients randomly allocated to other postoperative adjunctive therapies. Finally, a considerable increase of scores for all domains of SF-36 was observed in all women at 12 months’ follow-up, independently by the treatment randomly assigned.

#### Qualitative Studies

The most recent study on dietary changes in endometriosis is a qualitative interview study in which semi-structured qualitative interviews were conducted with 12 patients with endometriosis who had made individual dietary changes aimed at decreasing their endometriosis symptoms. The patients were asked about the dietary changes they made and changes in symptom severity after being recruited from two Swedish endometriosis support forums on the internet. The participants experienced an increase in well-being and a decrease in symptoms following their dietary and lifestyle changes. They also felt that the dietary changes led to increased energy levels and a deeper understanding of how they could affect their health by listening to their body’s reactions. No objective pain measurements were performed [51].

### Quality and Publication Bias Assessment in Human Studies

One RCT in this systematic review was assessed as a low risk bias study. It is, however, worth mentioning that with the inclusion of only 20 patients per group this trial was substantially underpowered to detect a significant difference. The second RCT included in the analysis was also assessed as a low-risk bias study. However, a high risk in attrition bias was given due to exclusion of lost to follow-up patients from the statistical analysis although this was the case for only 5.1% of the patients.

None of the observational studies were assessed as “low risk” of bias studies since they all received at least one high or moderate risk of bias in the pre-specified criteria according to the QUIPS tool. The only study receiving no high-risk bias assessment in any of the QUIPS categories was the study by Ott et al. [48]. The study of Vennberg et al. [51] was assessed as a high-risk bias study due to many methodological shortcomings. Participants were asked to talk freely about their changed nutritional habits; however, these were not systematically described. Moreover, the outcome of the study was not provided and endometriosis-associated symptoms were not quantified. The statistical analysis and reporting in the study by Moore et al. [49] received a high-risk bias rating because only the proportion of patients with “response” to the diet but not their VAS scores was presented. Signorile et al. did not report the number of participants lost to follow-up and the data were presented as percentage of patients with VAS score > 5. The study by Mier-Cabrera J. et al. [46] received a high-risk bias rating in study confounding since patients were assigned to the dietary intervention or normal diet according to the previous vitamin intake measurements so that the case groups was substantially different and not comparable to the control group. Moreover, only laboratory markers and no direct endometriosis-associated outcomes were measured which lowers the interpretability of the results. Finally, the study by Borghini et al. [47] and Marziali et al. [50] experienced a dropout rate of approximately 30%. The statistical analysis and reporting risk were deemed high since no intention to treat analysis was performed.

### Animal Studies

The 12 included animal studies were published between 2007 and 2018. All of them used a mouse model for endometriosis. Six studies performed the method of autotransplantation of uterine tissue to peritoneum [53–58], three studies used endometrial tissue from endometriosis-free women and implanted it to the abdominal cavity [59–61], one study used eutopic endometrial tissue from women with endometriosis [62] and two other studies transplanted the uterus from a donor mice [63, 64]. In two studies, the mice underwent overectomy [56, 60]. The study interventions were either dietary supplementations or caloric intake modifications. The detailed study characteristics are presented in Table 4 and the risk of bias assessment in Table 5.

**Table 4:** Characteristics of the animal studies

Author	Country	Study design	Intervention group	Control group	Dietary intervention (route of administration)	Results	Conclusions
Dietary supplementation							
Mariani et al., 2012 [57]	Italy	Controlled Trial	70	70	Elocalcitol (p.o.)	↓ lesion weight by 70% ↓ capacity of endometrial cells adherence to collagen inhibition of macrophage and inflammatory cytokine secretion	Elocalcitol inhibits endometriosis lesions development.
Akyol et al., 2015 [58]	Turkey	RCT	8 (Vit. D) 9 (omega-3)	*n* = 9	Vit. D (i.p.) Omega-3 (i.p.)	- Omega-3 led to ↓ lesion volume ↓ IL-6, TNF-alpha, VEGF levels - Vitamin D led to ↓ IL-6 levels	Omega-3 induced the regression of endometriosis implants in contrast to Vit. D.
Attaman et al., 2014 [56]	USA	Controlled trial	NA	NA	Endogenous omega-3 (p.o.)	↓ IL-6, Cox-2, phosphohistone 3 expression ↑ VEGF expression	Omega-3 levels play a role on the establishment of endometriosis-like lesions, influencing immune, angiogenic and proliferative aspects of endometriosis.
Herington et al., 2013 [59]	USA	RCT	9	NA	Fish oil (p.o.)	↓ adhesions ↓ collagen deposition	Supplementing fish oil reduces postsurgical adhesions related to endometriosis.
Xu et al., 2008 [62]	China	RCT	10 (Epigallocatechingallate) 10 (Vitamin. E)	n = 10	Epigallocatechin gallate (EGCG) i.p. Vitamin E (i.p.)	EGCG led to ↓ size of endometriotic lesions and glandular epithelium ↓ angionesis ↑ VEGFA, NF-κB, mitogen activated protein and mRNA levels of kinase 1	EGCG leads to inhibition of development of experimental endometriosis. No effects were noticed with Vitamin E supplementation.
Netsu et al., 2007 [54]	Japan	RCT	9 omega-3 9 omega-6	NA	Omega-3 (p.o.) Omega-6 (p.o.)	Omega-3 led to ↑ interstitium thickening ↓ inflammation ↓ mRNAs of interleukin-1b, metalloproteinases, prostaglandin E synthase, interleukin-1r and nuclear factor (NF)-kB	EPA supplementation is a considerable strategy for endometriosis treatment.
Bruner-Tran et al., 2010 [60]	USA	RCT	20	16	Resveratrol (p.o.)	↓ number of endometrial implants ↓ invasiveness of human endometrial tissue	Reservatol inhibits the development and reduces the invasiveness of endometriosis.
Rudzitis-Auth et al., 2012 [53]	Germany	Controlled Trial	8	8	Xanthohumol (p.o.)	↓ size of endometriosis lesions at day 28 ↓ phosphoinositide 3-kinase protein ↓ vascularization	Xanthohumol inhibits the development of endometriotic lesions in mice, avoiding side reproductive organs damages.
Agostinis et al., 2014 [61]	Italy	RCT	7	9	N-Acetyl cystein, alpha-lipoic acid, bromelain (p.o)	↓ number of endometriotic cysts	The combination of N-acetyl cystein, alpha-lipoic acid, Bromelain could reduce the number of endometriosis lesions.
Park et al., 2018 [55]	Republic of Korea	RCT	15	15	Quercetin (i.p.)	↓ mRNA Ccnd1 expression ↑ apoptosis of VK2/E6E7 and End1/E6E7 cells	Quercetin inhibited the proliferation and induced the cell cycle arrest and cell apoptosis.
Calorie intake changes							
Heard et al., 2016 [64]	USA	RCT	9	7	High-fat diet (45% fat kcal)	↓ stromal estrogen receptor 1 isoform ↓ progesterone receptor expression ↑ F4/80-positive macrophage infiltration ↑ stromal proliferation ↑ expression of proinflammatory and prooxidative stress pathway genes ↑ TNFa-levels in peritoneal fluid ↑ local and systemic redox status	High-fat diet intake increased the number of the endometriosis lesions while no alterations were observed in weight gain, ovarian function and insulin resistance.
Yin et al., 2018 [63]	China	RCT	10	10	Caloric restriction (30% less)	↓PI3K/AKT/mTOR signaling, ↑ SIRT1 and AMPK signaling -promoting autophagy ↓local estrogen production, cellular proliferation, angiogenesis and fibrogenesis.	Caloric restriction (before the induction of endometriosis) leads to a much reduced lesion weight.

*p.o.*, per os; *i.p*. intraperitoneal injection

**Table 5:** Quality assessment of animal studies (SYRCLE tool)

	Sequence generation (selection)	Baseline characteristic (selection)	Allocation concealment (selection)	Random housing (performance)	Blinding (performance)	Random outcome assessment (detection)	Blinding (detection)	Incomplete outcome data (attrition)	Selective outcome reporting (reporting)	Other biases
Dietary supplementation										
Mariani et al., 2012	U	L	U	U	U	U	U	L	L	L
Akyol et al., 2015	U	L	U	L	U	U	U	L	L	L
Attaman et al., 2014	U	L	U	L	U	U	L	L	L	L
Herrington et al., 2013	U	L	U	L	U	U	U	L	L	L
Xu et al., 2008	U	L	U	L	U	U	L	L	L	L
Netsu et al., 2007	U	L	U	L	U	U	U	L	L	L
Bruner-Tran et al., 2010	U	L	U	U	U	U	U	L	L	L
Rudzitis-Auth et al., 2012	U	L	U	L	U	U	U	L	L	L
Agostinis et al., 2014	U	L	U	U	U	U	U	L	L	L
Park et al., 2018	U	L	U	L	U	U	U	L	L	L
Calorie intake changes										
Heard et al., 2016	U	L	U	U	U	U	U	L	L	L
Yin et al., 2018	U	L	U	L	U	U	U	L	L	L

*L*, low risk; *H*, high risk; *U*, unclear risk

## Discussion

This is the first systematic review of the effect of dietary interventions in patients with endometriosis. Given the high possibility of these patients to seek complementary therapies including dietary modulations [65–67] as well as the recently established multidisciplinary programs involving dieticians in most endometriosis centers [68], it becomes urgent that the up-to-date evidence to guide clinical practice is presented. Although a meta-analysis was impossible due to the high heterogeneity of the interventions and measured outcomes, the study provides a thorough overview of the published studies on this topic. The review identified nine human studies all of which assessed a different dietary intervention with most of them finding a positive effect on endometriosis. However, all studies are characterized by moderate and/or high-risk bias limiting the validity of the results.

### Results in the Context of What is Known

The association between vitamin D levels and endometriosis remains unclear to date [69]. In the current review, the only study showing no positive effect of the dietary intervention on endometriosis was an RCT exploring vitamin D supplementation in patients with unknown serum vitamin D levels. Another RCT with the same sample size including patients with primary dysmenorrhea and vitamin D insufficiency but no endometriosis has previously shown a positive effect of vitamin D on pain (standardized mean difference − 1.02; 95% CI − 1.9 to 0.14; *P* = 0.024) [70]. A significantly greater mean decrease in pain has been also observed in a systematic meta-analysis evaluating vitamin D supplementation in patients with several pain disorders (mean difference − 0.57; 95% CI − 1.00 to − 0.15; *P* = 0.007) [71]. It is however obvious that the results of these studies cannot be extrapolated to the general endometriosis population so that vitamin D supplementation cannot be currently recommended in patients with endometriosis unless there is a documented vitamin D deficiency. We identified one trial protocol aiming to investigate the supplementation of vitamin D3 in adolescent girls with endometriosis, which was completed in 2016 but since then no data were published [72].

Three studies included in the current review investigated the role of an antioxidant supplementation. This included specific vitamins, fish oils, and mineral salts with different protocols in each study. A significant reduction of symptoms was observed in two of the studies one of which was a randomized controlled trial [44, 45]. The third study only measured blood oxidative stress markers, which were found to be decreased after dietary intervention [46]. Finally, one study conducted in the USA was published only as an abstract in 2003 and thus excluded from the qualitative synthesis [73]. Nevertheless, the study investigated the effect of vitamin E and vitamin C supplementation for 2 months on inflammatory markers in women with endometriosis. A decrease in all the inflammatory markers (MCP-1, RANTES, IL-6) in the peritoneal fluid was seen according to the results in the abstract. Overall, based predominantly on the available RCT, it seems that this type of dietary interventions may have the potential to ameliorate endometriosis-associated pain. Currently, there are four registered protocols for clinical trials on this type of dietary supplementations [74–77].

The study by Ott et al. [48] could also be included in the above category since the suggested Mediterranean diet has well-known antioxidant effects. However, the Mediterranean diet does not involve just the supplementation of certain antioxidants, but rather a collection of eating habits and may thus improve endometriosis-associated pain via additional mechanisms. Fish as well as extra virgin olive oils have been shown to exert anti-inflammatory effects. Specifically, extra virgin olive oil, which contains the substance oleocanthal, displays a similar structure to the molecule ibuprofen, and both take effect via the same mechanism, i.e., cyclooxygenase inhibition. Moreover, the increased amount of fibers provides a eupeptic effect while foods high in magnesium could prevent an increase in the intracellurar calcium level and advance the relaxation of the uterus [78]. Taking into account the lack of risks or side effects even after long-term lifetime adherence to this diet and the possible other general health benefits [20, 22, 23] clinicians may suggest this type of dietary intervention to patients with endometriosis who wish to change their nutritional habits. Two protocols for clinical trials are currently registered with the one investigating the effect of Mediterranean diet and physical activity [79] and the other the Alternative Healthy Eating Index diet [80] in patients with endometriosis.

The other category of dietary interventions in this review consists of diets excluding or reducing specific substances such as gluten-free, low-Ni, and low-FODMAP diet. Two studies included only patients with endometriosis and gastrointestinal symptoms [47, 49] while the third one patients with endometriosis in general [50]. All studies showed an improvement of symptoms in at least 70% of the patients adhering to the diet. However, interestingly, in the gluten-free and low-Ni diet, approximately 30% of the patients did not adhere to the diet while no intention-to-treat analysis was performed. In the low-FODMAP diet, a higher adherence was observed maybe due to the shorter time of the intervention (4 weeks) or the exclusive inclusion of patients with irritable bowel syndrome. Because of FODMAPs’ characteristic of insufficient absorption and their consecutive fermentation by bacteria, their gas production tends to cause abdominal intestinal extension that may lead to pain and modification of gut motility. Diseases like irritable bowel syndrome and endometriosis come along with visceral hypersensitivity, implementing the hypothesis of symptom-reduction after sticking to a low-FODMAP diet [81, 82]. This is very important given the high prevalence of gastrointestinal-related symptoms and co-morbidities in patients with endometriosis [83, 84]. Interestingly, the low-FODMAP diet includes not only a low-Ni diet, but also a low-lactose and a low-gluten diet, thus covering the abovementioned diets and a large spectrum of high-prevalence pathologies, such as lactose intolerance and non-celiac gluten sensitivity. It is therefore possible to obtain clinical benefits from a low-FODMAP diet, even if at the cost of probably not necessary dietary exclusions. Prospective studies with low-FODMAP diets in patients with endometriosis with clear description of their symptoms and co-morbidities are needed to elucidate this issue. We identified one registered protocol for a pilot clinical trial on the effects of low-FODMAP diet on endometriosis-related gastrointestinal symptoms, which should be completed by February 2018 but not yet published [85].

The aim of this review was to evaluate the role of dietary interventions in patients with previously diagnosed endometriosis. Many studies on the effects of dietary supplements on dysmenorrhea exist though. Dysmenorrhea is the most common symptom in endometriosis but it is also highly non-specific so that dysmenorrhea does not necessitate the existence of endometriosis. Nevertheless, the existing evidence is worth mentioning. The most recent Cochrane systematic review concluded that there is very limited evidence of effectiveness for fenugreek (MD − 1.71 points; 95% CI − 2.35 to − 1.07; one RCT, 101 women), fish oil (MD 1.11 points; 95% CI 0.45 to 1.77; one RCT, 120 women), fish oil plus vitamin B1 (MD − 1.21 points; 95% CI − 1.79 to − 0.63; one RCT, 120 women), ginger (MD − 1.55 points; 95% CI − 2.43 to − 0.68; three RCTs, 266 women; OR 5.44; 95% CI 1.80 to 16.46; one RCT, 69 women), valerian (MD − 0.76 points; 95% CI − 1.44 to − 0.08; one RCT, 100 women), vitamin B1 (MD − 2.70 points; 95% CI − 3.32 to − 2.08; one RCT, 120 women), zataria (OR 6.66, 95% CI 2.66 to 16.72; one RCT, 99 women), and zinc sulphate (MD − 0.95 points; 95% CI − 1.54 to − 0.36; one RCT, 99 women) [86]. In the same Cochrane review, one RCT was included evaluating melatonin in patients with endometriosis [87]. The study authors reported that melatonin reduced dysmenorrhea and daily pain scores. However, this study was excluded from our review since the administration of melatonin cannot be considered as a dietary intervention but more as a hormonal treatment.

The animal studies included in this review present promising results that need confirmation in human studies. Interestingly, a high-fat diet intake increased the number of the endometriosis lesions [64] while caloric restriction leaded to a reduced endometriosis lesion weight [63]. Moreover, omega-3 polyunsaturated fatty acids (PUFA) were shown to induce the regression of endometriosis [54, 56, 58], which is partially in line with the human studies. Finally, several natural components found in plants such as resveratrol (natural phenol found in grapes and red wine) [60], quercetin (a plant flavonol) [55], xanthohumol (found in beer) [53], and epigallocatechingallate (found in green tea) [62] seem to inhibit endometriosis. However, due to the way of inducing endometriosis in animals, the route of nutrition administration in some studies and the lack of symptom measurement, all of which do not reflect the reality in humans, the results of the animal studies cannot be extrapolated to changes in the patients’ diet.

### Implications for Practice

**Fig. 2:**
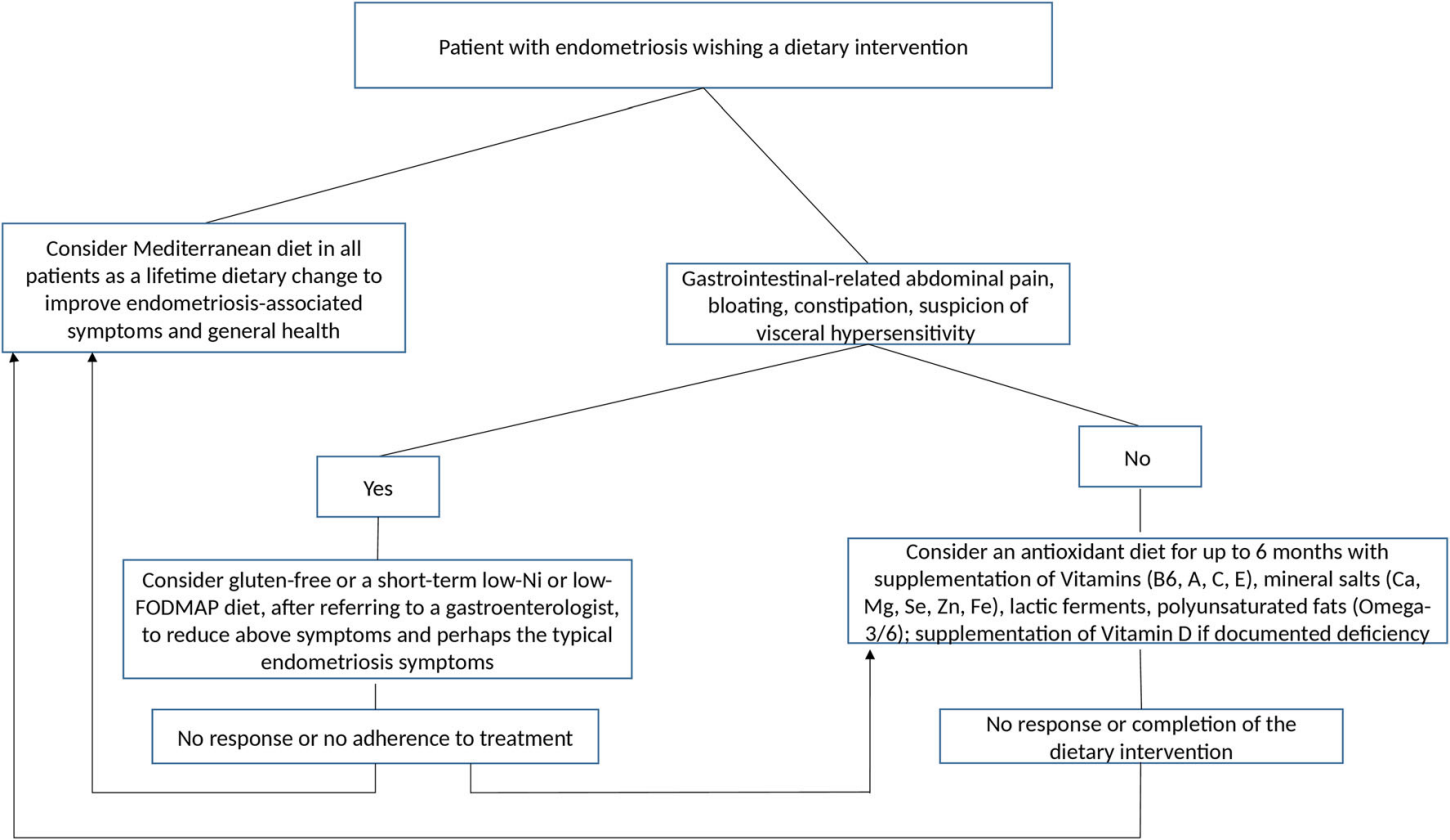
A suggested approach to dietary interventions in endometriosis

As presented above, only a small number of studies to date have examined dietary modifications to treat endometriosis and endometriosis-associated symptoms. A high study heterogeneity was identified and all studies are characterized by moderate or high-risk bias. Although most studies reported a positive effect of the intervention on endometriosis, we could not clearly identify certain subcategories of patients, which are more likely to benefit from a dietary intervention nor did we identify specific dietary interventions, which ameliorate certain endometriosis-associated symptoms. More and higher quality original studies are urgently needed to enable safe conclusions on this topic. Until then, we suggest a possible clinical approach to the patient with endometriosis seeking a dietary change to treat her symptoms (Fig. 2). Based on the promising results of the prospective before-after study by Ott et al. [48] as well as considering the well-known health benefits and no risks of Mediterranean diet, physicians may suggest this diet as a long-term dietary change. However, due to the difficulty in adhering to Mediterranean diet related to the costs, cooking habits, and personal daily life, a supplementary antioxidant diet may be considered for up to 6 months based on the RCT by Sesti et al. [44]. A longer than 6 months intake of dietary supplements cannot be suggested due to the unclear long-term safety of such interventions [88–90]. On the other hand, patients with gastrointestinal-related abdominal pain, bloating, and constipation as well as suspicion of visceral hypersensibility may benefit from a gluten-free, low-Ni, or low-FODMAP diet. However, these diets may also be related to lower adherence due to the associated financial burden and inherent difficulties. Moreover, the safety of a long-term use of low-Ni has not been established while a long-term low-FODMAP diet can have negative effects on gut microbiota [91, 92]. The start of these diets should be decided in a multidisciplinary approach after gastrointestinal evaluation in order to exclude other pathological diagnoses.

### Strengths and Limitations

A limitation of the review is that only studies reported in English were included, and there may be relevant studies in other languages, which were missed. The included studies varied significantly in the type of dietary intervention, the follow-up and the outcome measurements while they also suffered from a non-negligible risk of bias so that it was impossible to perform a meta-analysis and difficult to draw a clear picture. These limitations are partially due to the inherent weaknesses of dietary intervention studies in general. However, by presenting and discussing the results of the studies thoroughly, a clinical approach, according to the available level of evidence, can be now suggested which might consist a roadmap for clinicians and patients. Finally, the originality of the study should be mentioned since it represents the first systematic presentation of the up-to-date evidence on the therapeutic role of dietary interventions in endometriosis. The study is expected to enhance the clinical decision-making in the rapidly evolving field of patient’s active participation in the treatment against their disease.

## Conclusions

Given that almost all studies in this review found a significant reduction of pain, dietary interventions appear promising to treat endometriosis-related symptoms. These results should however be treated with caution due to the limited number of available studies, the high heterogeneity and the high risk of bias. Proper counseling prior to the start of such treatments and where appropriate in a multidisciplinary setting including gastroenterologists and/or dieticians is warranted. Furthermore, well-designed randomized controlled trials are needed to accurately determine the short-term and long-term effectiveness and safety of dietary interventions.
